# Draft Genome Sequence and Annotation of Priestia aryabhattai Strain BD1 Isolated from a Dye Sediment

**DOI:** 10.1128/mra.01175-22

**Published:** 2023-01-09

**Authors:** Imade Y. Nsa, Blessing M. Omolere

**Affiliations:** a Department of Microbiology, Faculty of Science, University of Lagos, Lagos, Nigeria; University of Maryland School of Medicine

## Abstract

The 5,079,658-bp genome of Priestia aryabhattai strain BD1 isolated from a dye waste sediment consists of 5,185 protein-coding genes, of which 790 were hypothetical proteins, 63 were RNAs, and 54 were pseudogenes. Putative genes involved in oxygen/redox potential sensing, dye degradation, metal toxicity, antibiotic tolerance, and benzoate metabolism were found.

## ANNOUNCEMENT

Priestia aryabhattai, a Gram-positive rod, formerly called Bacillus aryabhattai (1), was first isolated from cryotube*s* (2). Recently, a *P. aryabhattai* isolate from chernozem soil was reported to degrade benzoate (3), while another strain from textile wastewater has azo dye degradation capabilities (4). Reactive azo dyes, the principal pigments used in textile industries, contain aromatic compounds. Improper disposal of commercial dyestuff effluents is an anthropogenic source of these compounds in the environment. A dye waste sediment sample was collected behind a textile company in Lagos, Nigeria, to explore for azo dye-degrading bacteria. To isolate indigenous bacteria, 10-fold serial dilutions of up to 10^−6^ of the sediment sample were prepared and grown on tryptic soy agar plates and incubated at 37°C for 18 h. Pure cultures of unique isolates that were screened from the total culturable bacteria were individually tested on minimal salts medium infused with Cibacron dyes—reactive blue 4% red (RBFR) and brilliant yellow 6% green (CYPGS) (5). The Cibacron dyes were provided by the textile company. *P. aryabhattai* was one of the four decolorizers selected for whole-genome sequencing.

Genomic DNA was isolated from cells of *P*. *aryabhattai* grown overnight at 37°C in tryptic soy broth (TSB) using the Zymo ZR Quick-DNA fungal/bacterial miniprep (5). The Illumina NextSeq 500 sequencing (2- by 150-bp long paired-end) was done at Quadram Institute Bioscience, Norwich, United Kingdom.

A DNA library was prepared using the Nextera XT index kit v2 (FC-131-2001 to 2004). The 2,388,150 trimmed reads with an average read length of 149 bp were obtained using the Trim Galore tool v0.6.5 (6) and assessed for quality using FastQC v0.11.9 (7). Genome assembly was done using SPAdes v3.13.3 (8) and assessed for quality using QUAST v5.0.2 (9, 10) and CheckM v1.0.18 (11). NCBI Prokaryotic Genome Annotation Pipeline (PGAP) v6.2 (12–14) was used to annotate the contigs deposited into GenBank and the tRNAs were called using Aragorn v1.2.41 (15). All tools were run using default parameters.

The genome completeness on CheckM was estimated to be 99.43%. The resulting genome was 5,079,658 bp in length with 70.02× genome coverage and 38.06% GC content. The largest of the 57 contigs was 539,845 bp, with an *N*_50_ of 136,567. PGAP called 5,302 genes consisting of 5,185 protein-coding genes (comprising 790 hypothetical proteins), 7 rRNAs, 6 noncoding RNAs (ncRNAs), 50 tRNAs, and 54 pseudogenes. However, Aragorn called 53 tRNAs and one transfer-messenger RNA (tmRNA).

Putative genes involved in the resistance to antibiotics and toxic substances (fosfomycin, arsenic, cobalt, and zinc), dye decolorization (multiple copies of flavin mononucleotide [FMN]-dependent NADH-azoreductases), and metabolism of aromatic compounds (benzoate), three Per-Arnt SIM (PAS) domain proteins, and several copies of PAS domain S-box proteins were present.

### Data availability.

The project data of *Priestia aryabhattai* BD1 are available under BioProject number PRJNA858057, BioSample number SAMN29663492, and SRA accession number SRP386072. This whole-genome shotgun project has been deposited at DDBJ/ENA/GenBank under the accession number JANIPC000000000. The version described in this paper is version JANIPC010000000.
